# Characterization of *Bathyarchaeota* genomes assembled from metagenomes of biofilms residing in mesophilic and thermophilic biogas reactors

**DOI:** 10.1186/s13068-018-1162-4

**Published:** 2018-06-19

**Authors:** Irena Maus, Madis Rumming, Ingo Bergmann, Kathrin Heeg, Marcel Pohl, Edith Nettmann, Sebastian Jaenicke, Jochen Blom, Alfred Pühler, Andreas Schlüter, Alexander Sczyrba, Michael Klocke

**Affiliations:** 1 Dept. Bioengineering, Leibniz Institute for Agricultural Engineering and Bioeconomy (ATB), Max-Eyth-Allee 100, 14469 Potsdam, Germany; 2 Center for Biotechnology (CeBiTec), Bielefeld University, Universitätsstrasse 27, 33615 Bielefeld, Germany; 3 Computational Metagenomics, Faculty of Technology, Bielefeld University, Universitätsstrasse 25, 33615 Bielefeld, Germany; 4 Biochemical Conversion Department, Deutsches Biomasseforschungszentrum gemeinnützige GmbH, Torgauer Straße 116, 04347 Leipzig, Germany; 5 Urban Water Management and Environmental Engineering, Faculty of Civil and Environmental Engineering, Ruhr University Bochum, Universitätsstraße 150, 44780 Bochum, Germany; 6 Dept. Bioinformatics and Systems Biology, Justus-Liebig University Gießen, Heinrich-Buff-Ring 58, 35392 Giessen, Germany

**Keywords:** *Archaea*, *Bathyarchaeota*, Biomass conversion, Anaerobic digestion, Biomethanation, Hydrolysis, Metabolic pathway reconstruction, Metagenome-assembled genomes, Genome binning

## Abstract

**Background:**

Previous studies on the *Miscellaneous Crenarchaeota Group*, recently assigned to the novel archaeal phylum *Bathyarchaeota*, reported on the dominance of these *Archaea* within the anaerobic carbohydrate cycle performed by the deep marine biosphere. For the first time, members of this phylum were identified also in mesophilic and thermophilic biogas-forming biofilms and characterized in detail.

**Results:**

Metagenome shotgun libraries of biofilm microbiomes were sequenced using the Illumina MiSeq system. Taxonomic classification revealed that between 0.1 and 2% of all classified sequences were assigned to *Bathyarchaeota.* Individual metagenome assemblies followed by genome binning resulted in the reconstruction of five metagenome-assembled genomes (MAGs) of *Bathyarchaeota*. MAGs were estimated to be 65–92% complete, ranging in their genome sizes from 1.1 to 2.0 Mb. Phylogenetic classification based on core gene sets confirmed their placement within the phylum *Bathyarchaeota* clustering as a separate group diverging from most of the recently known *Bathyarchaeota* clusters. The genetic repertoire of these MAGs indicated an energy metabolism based on carbohydrate and amino acid fermentation featuring the potential for extracellular hydrolysis of cellulose, cellobiose as well as proteins. In addition, corresponding transporter systems were identified. Furthermore, genes encoding enzymes for the utilization of carbon monoxide and/or carbon dioxide via the Wood–Ljungdahl pathway were detected.

**Conclusions:**

For the members of *Bathyarchaeota* detected in the biofilm microbiomes, a hydrolytic lifestyle is proposed. This is the first study indicating that *Bathyarchaeota* members contribute presumably to hydrolysis and subsequent fermentation of organic substrates within biotechnological biogas production processes.

**Electronic supplementary material:**

The online version of this article (10.1186/s13068-018-1162-4) contains supplementary material, which is available to authorized users.

## Background

The bioconversion of biomass to biogas by anaerobic digestion (AD) is a process commonly found in nature which is performed by highly diverse and dynamic microbial communities. In the break-down cascade of macromolecular compounds, methanogenesis is the last step conducted exclusively by methanogenic *Archaea* of the phylum *Euryarchaeota*.

The structure and development of biomass-degrading microbial communities residing in biogas plants and, in particular, of the participating methanogenic archaeal species have been intensively studied [1–4]. Hydrogenotrophic *Archaea* utilizing H_2_ and CO_2_ often dominate the archaeal sub-communities in biogas-producing systems, while the acetoclastic and methylotrophic methanogens are less abundant [3, 5]. H_2_/CO_2_ as well as acetate and other volatile fatty acids are provided by various fermentative bacteria predominantly affiliated with the classes *Clostridia* and *Bacteroidia* [2, 4, 6]. However, metagenome studies addressing biogas-producing microbial community characterization reported on a huge fraction of sequences that cannot be classified to higher taxonomic ranks suggesting that, for the most part, the microbial species present in biogas microbiomes are so far unknown [4, 7].

On the other hand, the non-cultivable fraction of biogas-producing microbial communities becomes accessible even by applying metagenome assemblies combined with binning methods enabling the identification of novel and, hence, metabolically uncharacterized species [8, 9]. Using this strategy, Evans and colleagues [10] were able to recover two metagenome-assembled genomes (MAGs, denominated as BA1 and BA2) of the phylum *Bathyarchaeota* from a deep aquifer habitat within the Surat Basin (Australia). The proposed phylum *Bathyarchaeota* of the domain *Archaea* represents an evolutionary diverse group of microorganisms (previously denominated as *Miscellaneous Crenarchaeotal Group*, MCG) supposed to be widespread in nature [11–13]. In particular, the organic-rich sediments of the White Oak River estuary (North Carolina, USA) were described to be abundant in uncultured *Archaea*, especially members of the phylum *Bathyarchaeota* [12, 14, 15]. Studies on *Bathyarchaeota* metabolic function in situ via stable carbon isotope probing of the sediment archaeal community suggested that they assimilate organic carbon sources including acetate, glycine or urea, or complex biopolymers such as lipids, proteins, and the algal lipid/pigment extract in their sediment habitat [16]. A recent study by He and colleagues [17] indicated that *Bathyarchaeota* also have the potential to fix inorganic carbon in the form of CO_2_ to produce acetate, an important substrate for other sediment residents such as methanogenic *Archaea* or heterotrophic *Bacteria*. Moreover, based on the metabolism reconstructed from the MAG datasets, Evans and colleagues [10] suggested that BA1 and BA2, originating from microbial biomass from filtered waters within the Surat Basin (Queensland, Australia), are capable of methylotrophic methanogenesis indicating that methane metabolism also may exist outside the phylum *Euryarchaeota.*

This study focusses exclusively on the identification of *Bathyarchaeota* members in exemplary biotechnological AD processes and the analysis of their putative role during biomethanation of crop biomass and residues. Since previous studies reported on the abundance of *Bathyarchaeota* in natural environments, it was also of importance for this study to determine the abundance of this archaeal group in biogas reactor systems and to analyze whether standard reactor operating parameters might affect their occurrence. For this purpose, the metagenomes of different biomass-degrading and biogas-producing biofilm microbiomes obtained from different mesophilic (37 °C) and thermophilic (55 °C) two-phase, two-stage laboratory-scale biogas reactor systems consisting each of hydrolysis fermenters and anaerobic filters were sequenced.

Metagenome assemblies followed by a binning approach resulted in the identification of five *Bathyarchaeota* MAGs which were further analyzed in detail. These MAGs represent the first *Bathyarchaeota* members that have been identified in biogas-producing reactor systems so far.

## Methods

### Set-up, operation, and sampling of biofilms from two-phase, two-stage laboratory-scaled biogas fermenter systems

Three laboratory-scaled experimental biogas fermenter systems were sampled. As inocula for fermenter start-up, digestates and/or process liquids from previous AD experiments were used. *System 1* was a thermophilic (55 °C) two-phase, two-stage reactor system consisting of an upflow anaerobic solid-state (UASS) reactor digesting wheat straw as sole substrate and a downstream packed bed anaerobic filter (AF) with working volumes of 39 and 30 L, respectively [18]. Samples for microbial DNA extraction and subsequent metagenome sequencing were taken from the wheat straw digestate in the UASS to obtain the digestate-attached cellulolytic/hydrolytic biofilm, at day 160 of reactor operation and an organic loading rate (OLR) of 8 g volatile substances (VS) L^−1^ day^−1^. *System 2* was constructed similar to *system 1* but with a working volume of 27 L for the UASS and 22 L for the AF [19]. UASS and AF were operated at 37 °C. In the UASS, maize silage was co-digested with straw at an OLR of 3.0 g_VS_ L^−1^ day^−1^. Samples were taken from the methanogenic biofilms on the surfaces of randomly selected polyethylene packings of the AF at day 72 of operation. *System 3* was constructed, operated, and sampled similar to *system 2* but in this case, the entire system was operated at 55 °C. Further details on reactor operation were provided as Additional file 1.

### Metagenome sequencing, assembly, and binning, and functional analyses of obtained MAGs

Total microbial community DNA was extracted from samples and stored at − 20 °C using the FastDNA™ Spin Kit for Soil (MP Biomedicals, USA) according to the manufacturer’s instructions. Metagenome shotgun libraries were constructed applying the TruSeq DNA PCR-Free Library Preparation Kit (Illumina) and sequenced on the Illumina MiSeq system utilizing the V2 kit chemistry (Illumina). Trimmed and quality controlled metagenome sequences were assembled with MEGAHIT [20] setting the ‘meta-sensitive’ option and a minimal contig size of 1000 bp. Mappings of the metagenome data sets onto the assemblies were performed applying bbmap from the BBTools package [21] and were further processed with SAMtools [22]. LCAs (lowest common ancestor) of the contigs were computed with MEGAN6 [23] and were used as taxonomic assignments. For abundance determination of the taxonomically assigned contigs, the transcripts per million (TPM) was computed based on the mapped sequencing reads per reactor system individually. Binning of the assemblies was performed on contigs with a minimal coverage of twofold applying MetaBAT with default parameters [24]. Contamination and completeness level of the identified *Bathyarchaeota* MAGs were assessed with CheckM [25] and acdc [26]. Obtained *Bathyarchaeota* MAGs were subsequently annotated applying the program Prokka [27] and uploaded into the software platform GenDB [28] for functional analysis. Detailed information on the subsequent bioinformatical analysis of obtained metagenome datasets, i.e., assembly, binning, and functional analysis, is provided as Additional file 1.

### Phylogenetic classification of the determined *Bathyarchaeota* MAGs in relation to members of the domain *Archaea*

To phylogenetically classify the *Bathyarchaeota* MAGs analyzed in relation to members of the domain *Archaea*, the phylogenetic trees based on concatenated single-copy-genes (SCG) and, in addition, on 16S rRNA genes were constructed. The SCG phylogenetic tree was built with 14 MAGs assigned previously to the phylum *Bathyarchaeota* or to MCG (Additional file 2), respectively, and 128 archaeal genomes selected from IMG/M [29]. The 16S rRNA gene based tree was generated using 16S rRNA gene sequences derived from selected archaeal representatives publically available in the SILVA database. Calculation of phylogenetic trees was accomplished applying RAxML version 8.1.16 [30] using the PROTGAMMALGF model with bootstrap calculations based on 1000 replicates and visualized with Phyl.io [31]. Further details are provided as Additional file 1.

## Results and discussion

### AD biofilm community structure

In contrast to aqueous process liquids, the surface-associated biofilms in anaerobic biogas reactors were rarely analyzed [32]. In this study, two different thermophilic (55 °C, *systems 1* and *3*) and one mesophilic (37 °C, *system 2*) laboratory-scale biogas fermenter systems digesting crop biomass were sampled to determine the presence of *Bathyarchaeota* members in the microbial biofilms. Due to the respective sampling site, the biofilm sampled from the surface of the digestate of *system 1* can be regarded as primarily cellulolytic/hydrolytic and acidogenic but also, although less pronounced, as methanogenic. In contrast, the biofilms established on the surface of the packings in the AFs of *systems 2* and *3* are assumed to predominantly represent the methanogenic phase.

To characterize the microbial community compositions in these biofilms, high-throughput whole microbial metagenome sequencing was performed. The three corresponding metagenome datasets generated on the Illumina MiSeq system comprise between 21,963,917 (*system 3*) and 25,209,139 sequence reads (*system 2*) (Additional file 3). Taxonomic classification of the biogas biofilm microbiome members based on metagenome sequence data was accomplished as described previously applying the LCA approach on taxonomically assigned contigs. In total 61,633 contigs for *system 1*, 170,682 contigs for *system 2* and 68,904 contigs for *system 3* were classified to be of prokaryotic origin; between 1.71 and 3.66% sequence reads assembled as contigs remained with no further taxonomic assignment (Additional file 3). For further analysis, metagenome sequences assigned to either the domain *Bacteria* or *Archaea* were taken as 100%.

**Fig. 1:**
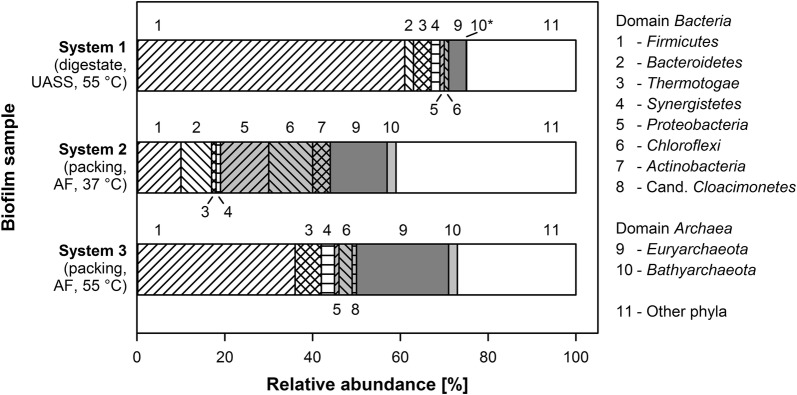
Relative abundance of the classified phyla present in the biofilms of the sampled thermophilic (*systems 1* and *3*) and mesophilic (*system 2*) biogas reactor systems. Analyses were performed on metagenomic data using the LCA (lowest common ancestor) algorithm implemented in MEGAN6 [23]. *UASS* upflow anaerobic solid-state reactor, *AF* anaerobic filter; *relative abundance 0.1%

Figure 1 represents relative abundances of classified sequences on phylum level of the analyzed biofilms. On higher taxonomic ranks, all taxonomic profiles showed the dominance of the domain *Bacteria* representing between 66 and 96% of all classified metagenome sequences. The most abundant phyla of the bacterial sub-communities in all biofilm samples are the *Firmicutes* (between 10 and 61%) followed by *Proteobacteria* (between 1 and 11%), *Chloroflexi* (between 1 and 10%), and *Thermotogae* (between 1 and 6%). The abundance of further phyla such as *Synergistetes* and Candidatus *Cloacimonetes* in thermophilic biofilms and *Bacteroidetes* and *Actinobacteria* in the mesophilic biofilm is in any case below 10%. As expected, these results support the importance of *Firmicutes* for anaerobic cellulolysis/hydrolysis, acidogenesis, and acetogenesis at mesophilic and thermophilic temperatures.

Taxonomic classification of the archaeal sub-communities revealed between 4 and 23% *Archaea* (Fig. 1). Members of the phylum *Euryarchaeota* are abundant in all microbiomes analyzed, representing between 4% (in the thermophilic cellulolytic/hydrolytic biofilm of *system 1*) and 21% (in *system 3*) of all classified metagenome sequences. Among the archaeal sequences obtained for biofilms of the reactor *systems 1*, *2* and *3*, 0.1, 2, and 2%, respectively, were classified to represent the phylum *Bathyarchaeota*. This is the first study, in which members of the newly proposed phylum *Bathyarchaeota* [10] were identified in biotechnological biogas-producing reactor systems digesting crop material.

### Phylogenetic affiliation of compiled *Bathyarchaeota* MAGs

To infer genetic potentials and possible functional roles of the detected so far unknown species assigned to the phylum *Bathyarchaeota*, metagenome assemblies followed by genome binning were applied. This approach enables the identification of new and uncharacterized genomes without the availability of reference database entries. The analysis resulted in the binning of a total of 78 MAGs that met the criteria of a minimum of 50% genome completeness and low contamination rates, i.e., less than 10%. All MAGs considered (Additional file 4) represent phyla shown in Fig. 1. Five of 78 MAGs belong to the phylum *Bathyarchaeota*. The MAGs ATB-1 (derived from the *system 1* dataset) and ATB-2, -3, and -4 (*system 3* dataset) were obtained for the thermophilic biofilms, and the MAG ATB-5 (*system 2* dataset) was determined for the mesophilic biofilm. The MAGs were estimated to be 65–92% complete as determined by the presence of single-copy marker genes (Table 1). The amount of contamination determined for the MAGs analyzed was low and might be caused by strain heterogeneity. Established MAGs’ sizes ranged from 1.1 to 2.0 Mb and featured GC contents from 42.17 to 48.94%. General genome features, e.g., assembly status, size, GC-content, and numbers of predicted genes, are summarized in Table 1.

**Table 1:** Statistics and general features of the *Bathyarchaeota* MAGs ATB-1, -2, -3, -4, and -5 analyzed in this study

Metagenome-assembled genome	ATB-1	ATB-2	ATB-3	ATB-4	ATB-5
Origin	Thermophilic biogas reactor system (55 °C)				Mesophilic biogas reactor system (37 °C)
	Digestate of *system 1*	AF of *system 3*	AF of *system 3*	AF of *system 3*	AF of *system 2*
	Cellulolytic/hydrolytic biofilm	Methanogenic biofilm	Methanogenic biofilm	Methanogenic biofilm	Methanogenic biofilm
Total length [bp]	2,038,732	1,574,857	1,495,994	1,083,171	1,914,325
Number of contigs	152	59	131	168	184
Largest contig	78,618	181,661	47,427	28,450	85,214
N50	18,903	37,126	13,707	8072	19,864
GC content [%]	48.45	45.80	45.36	48.94	42.17
Protein-coding genes	2279	1685	1709	1294	2042
Hypothetical proteins	873	597	671	501	832
rRNA genes	3 (16S-23S-5S)	n.d.	n.d.	n.d.	n.d.
tRNA genes	39	34	22	15	27
Completeness^a^	88.79	92.06	82.28	64.83	86.30
Contamination^a^	5.61	4.28	3.74	4.43	5.14

*AF* anaerobic filter, *n.d.* not determined

^a^Completeness and contamination were estimated by [25]

**Fig. 2:**
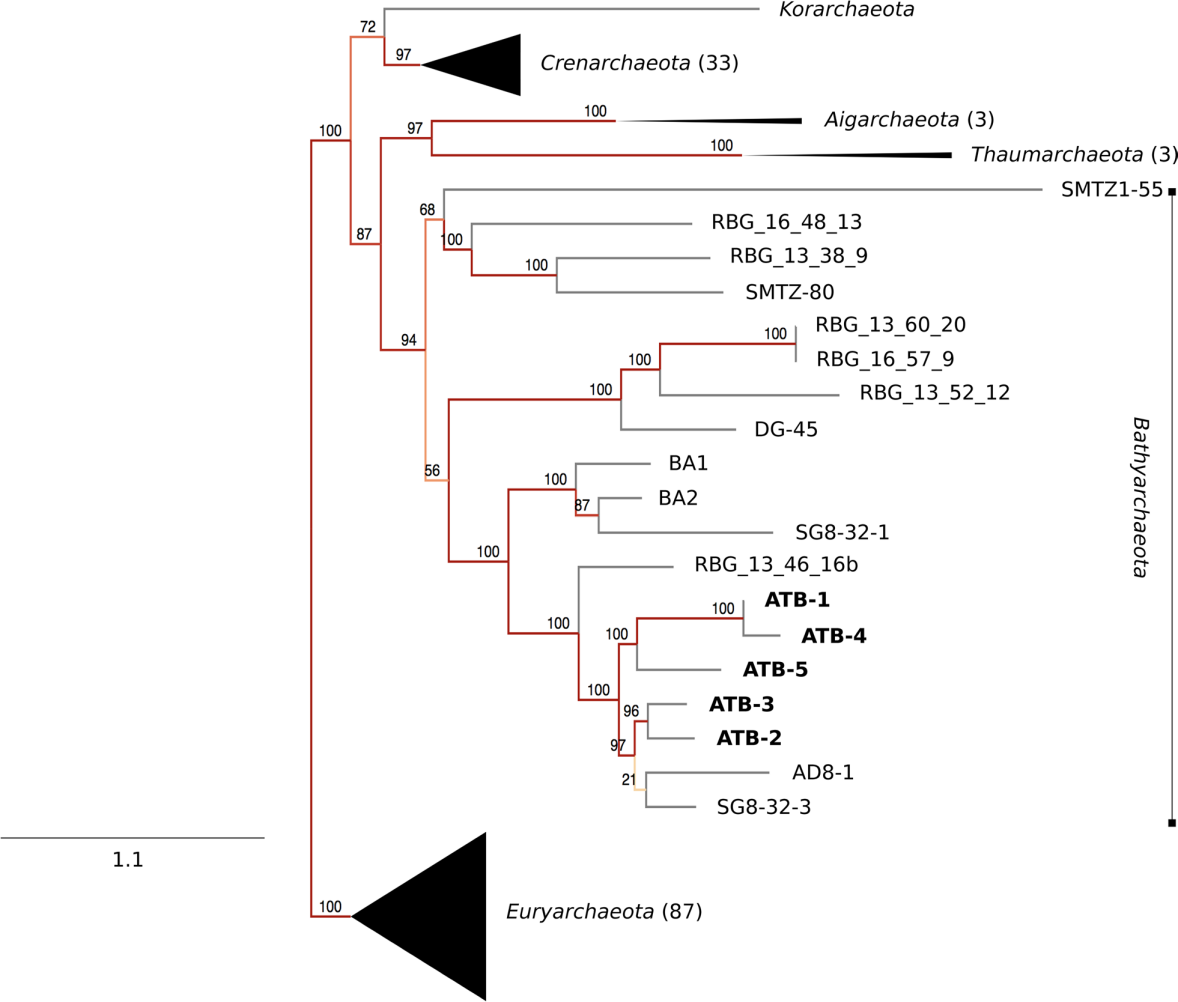
Phylogenetic classification of the *Bathyarchaeota* MAGs ATB-1, -2, -3, -4, and -5 originating from thermophilic and mesophilic biogas-producing microbial communities. The phylogenetic tree is based on all core genes of the selected strains and MAGs from the phyla *Euryarchaeota*, *Korarchaeota*, *Crenarchaeota*, *Aigarchaeota*, *Thaumarchaeota*, and *Bathyarchaeota* as determined by means of phylosift [46, 47] and RAxML version 8.1.16 [30]. Red-colored lines indicate a 100% support, orange tones accordingly less. BA1, BA2: MAGs determined for samples from coal-bed methane wells within the Surat Basin, Australia [10]; RBG_16_48_13, RBG_13_60_20, RBG_13_38_9, RBG_13_46_16b, RBG_16_57_9, RBG_13_52_12: MAGs determined for groundwater and sediment samples from an aquifer adjacent to the Colorado river near Rifle, USA [35], SG8-32-1, SMTZ-80, DG-45, AD8-1, SG8-32-3, SMTZI-55: MAGs determined for sediment core samples of the White Oak river, USA [34, 48–50]; further details on the MAGs are given in Additional file 2. The *Euryarchaeota* was chosen as outgroup. The bar represents the scale of sequence divergence

To determine the phylogenic affiliation of the five MAGs recovered from the metagenome data, SCG encoded gene products were compared to orthologous proteins of other members of the domain *Archaea* (Fig. 2). The resulting phylogenetic tree showed separation of the analyzed MAGs from other archaeal phyla included in this analysis, namely the *Euryarchaeota*, *Korarchaeota*, *Crenarchaeota*, *Aigarchaeota*, and *Thaumarchaeota*. Furthermore, the position of newly identified MAGs in the phylogenetic tree supports their affiliation to the phylum *Bathyarchaeota*.

Furthermore, the SCG based phylogenetic tree points to the closer relatedness of MAGs ATB-1 and MAG ATB-4 among the five analyzed MAGs. Hence, average nucleotide sequence identities (ANI) [33], suitable for species demarcation, were calculated between all MAGs analyzed (Additional file 5). MAGs ATB-1 and -4 showed an ANI value of 99.5%, indicating that these two members belong to the same species, whereas the remaining MAGs featured ANI values below 97% representing the species boundary [33]. However, it must be noted that the MAG ATB-4 only features a completeness of 65%. Moreover, it represents the smallest *Bathyarchaeota* MAG among the analyzed bins. Therefore, the statement about its species affiliation remains uncertain.

Interestingly, the *Bathyarchaeota* MAGs determined in this study cluster with the MAGs AD8-1 and SG8-32-3 originating from sediment cores of the White Oak river [34]. In contrast, they are separated from the MAGs BA1 and BA2 from a deep aquifer [10], SG8-32-1 (White Oak river habitat, [34] and RBG_13_46_16b (aquifer adjacent to the Colorado river [35]. Together with the *Bathyarchaeota* members AD8-1 and SG8-32-3, the MAGs obtained in this study build their own phylogenetic clade and revealed differences to the other recently published MAGs for MCG members. These results were confirmed by a 16S rRNA gene-based phylogenetic tree (Additional file 6), computed with sequences of archaeal members from the SILVA database and the 16S rRNA gene sequences from ATB-1.

### Pathways for carbohydrate metabolism present in the compiled *Bathyarchaeota* MAGs

The five *Bathyarchaeota* MAGs determined for the microbial biofilms residing in mesophilic and thermophilic biogas reactors were compared using the EDGAR software [36] in order to calculate the set of MAG-specific and shared protein-coding genes. The core genome of the MAGs analyzed appears to be small, including on average less than 26% of the genes of each MAG. This analysis revealed 338 orthologous genes shared by all of the analyzed MAGs (Fig. 5). These findings illustrate a large degree of genomic diversity in this *Bathyarchaeota* group. However, taking into account that ATB-4 represents the smallest of the analyzed *Bathyarchaeota* MAGs (65% completeness), an overestimation or on the contrary an underestimation of the genetic diversity in this group is most likely.

**Fig. 3:**
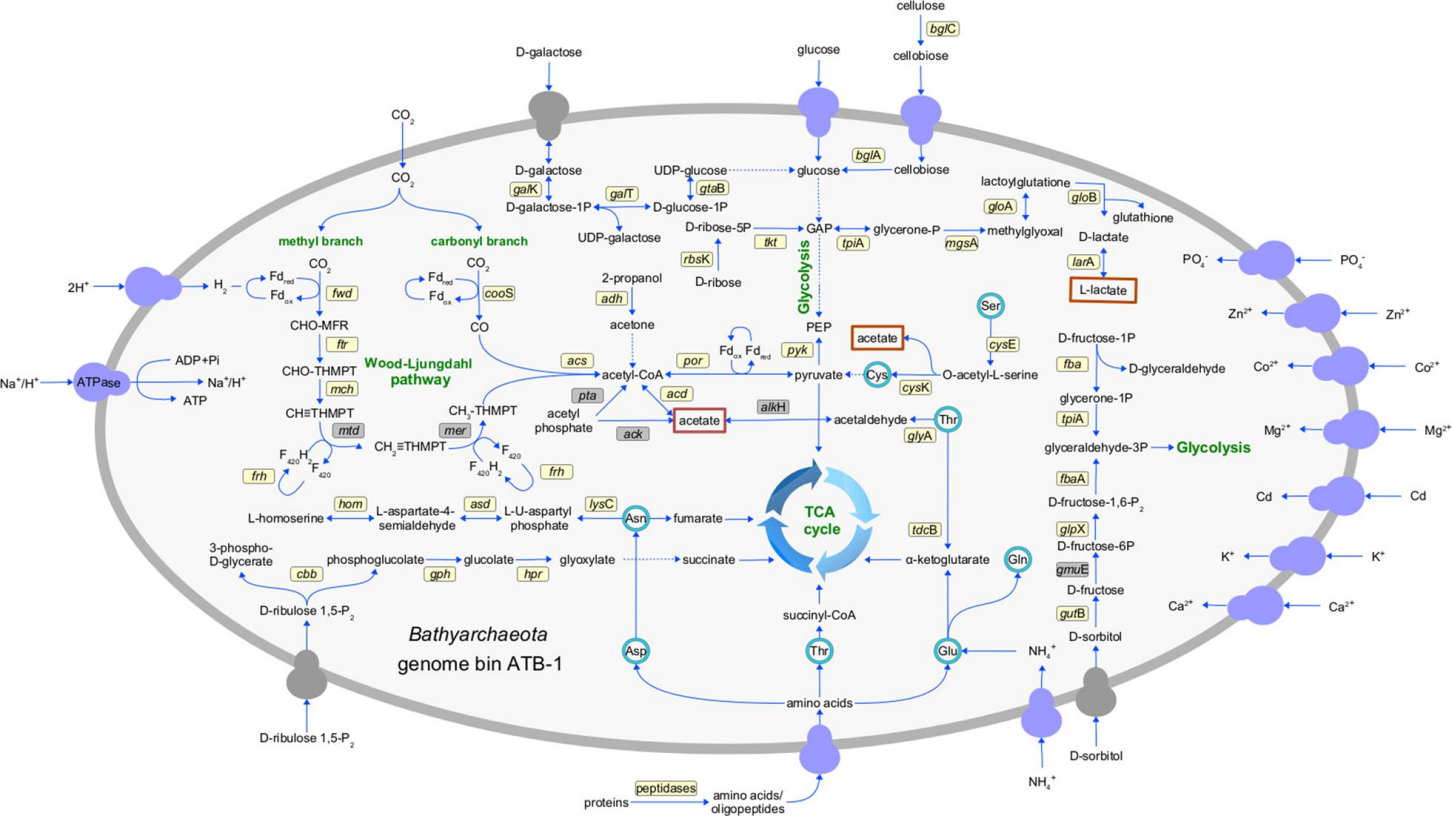
Metabolic reconstruction of central carbon metabolism pathways in *Bathyarchaeota* MAG ATB-1. Predicted metabolic pathways for conversion of carbohydrates, proteins and amino acids into acetate, lactate, and tricarboxylic acid intermediates are presented. Determined genes are shown in yellow boxes, determined transport systems are displayed in blue, and missing but presumed transport systems are indicated by gray coloring. Central metabolic pathways are highlighted in green. The identification numbers (locus tags) of MAG ATB-1 genes included in this analysis are listed in Additional file 7

To infer the functional roles of *Bathyarchaeota* MAGs originating from the sampled biofilms of mesophilic and thermophilic biogas reactor systems, metabolic reconstructions were done focusing on fermentation pathways represented in the KEGG database (Additional file 7). In Fig. 3, an overview of the major carbon compound utilizing metabolic pathways is exemplary given for MAG ATB-1, which is the largest MAG determined in this study.

**Fig. 4:**
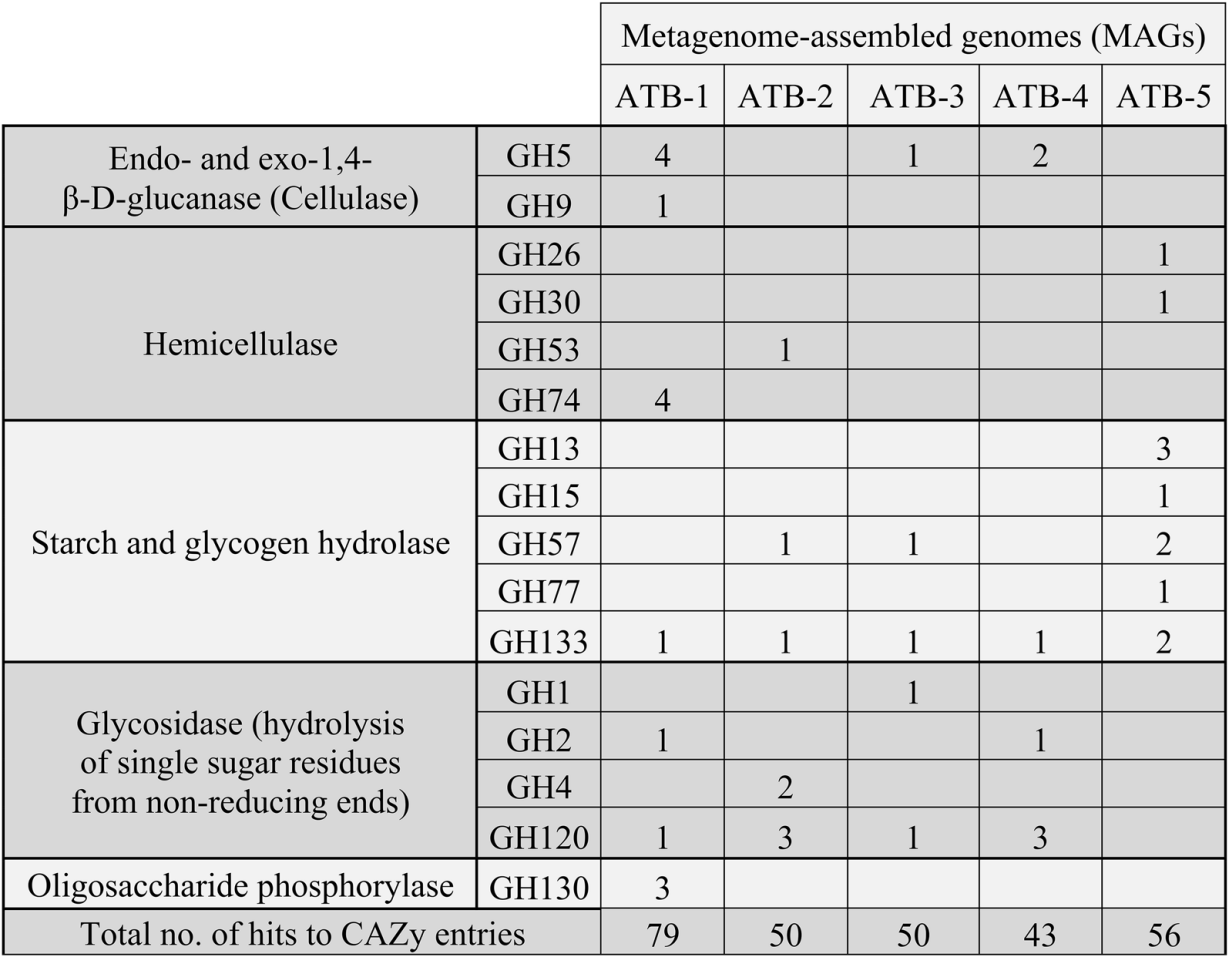
Diversity of genes encoding carbohydrate-active enzymes (CAZymes) predicted to be involved in hydrolysis and/or rearrangement of glycosidic bonds for the *Bathyarchaeota* MAGs ATB-1, -2, -3, -4, and -5. The screening for the presence of CAZymes was accomplished applying the Hidden-Markov-Model (HMM)-based Carbohydrate-active-enzyme Annotation database dbCAN [37]. The numbers of genes belonging to a corresponding glycosyl hydrolase (GH) family are given in the fields

Genomic profiling of the *Bathyarchaeota* MAGs and identification of genes encoding carbohydrate-active enzymes by means of the CAZy (Carbohydrate-Active-enZYmes) Database annotation web-server dbCAN [37] showed that all five MAGs have the genetic potential to import and utilize different carbohydrates including cellulose, cellobiose, galactose, glucose, ribose, and, additionally, sorbitol with ATB-1 showing the highest number of hits to CAZy entries (Fig. 4). Decomposition of these compounds results in metabolites that can enter the glycolysis pathway, which is completely encoded in all *Bathyarchaeota* MAGs analyzed. This indicates a metabolism based on carbohydrate fermentation as it was previously proposed for *Bathyarchaeota* members originating from other environments [34, 38].

Biomasses such as maize and straw (‘energy crops’) used for AD in biogas plants of this study represent plant materials rich in long-chained carbohydrates such as cellulose, hemicellulose, xylan, and starch, among others, but additionally comprise considerable amounts of proteins. Therefore, *Bathyarchaeota* MAGs were screened for genes encoding enzyme involved in protein, peptide, and amino acid transport and metabolism. The genetic repertoire of the MAGs analyzed also uncovered their potential to utilize proteins and amino acids as growth substrates which is in line with previous findings [10, 34]. In this context, all genes encoding enzymes involved in asparagine, aspartate, alanine, threonine, glutamate, glutamine, serine, and homoserine degradation into tricarboxylic acid (TCA) cycle intermediates and, additionally, pyruvate were identified (Additional file 7). The evidence for genes for carbohydrate, protein, and amino acid uptake and degradation indicate that *Bathyarchaeota* from the analyzed biogas plant share a heterotrophic metabolism. As it was previously postulated for *Bathyarchaeota* from the White Oak River sediments [34], this metabolism is primarily based on complex carbohydrates as carbon source augmented by utilization of peptides and amino acids.

Furthermore, the gene repertoire of the *Bathyarchaeota* MAGs revealed a set of genes, which were assigned to the Wood–Ljungdahl (WL) pathway. This pathway plays an important role in carbon fixation and acetate utilization in acetogens and methanogenesis in methanogenic *Archaea* and is characterized by two branches, namely the Western/Carbonyl and the Eastern/Methyl branch [39]. The reaction cascades of both WL branches can proceed in forward and reverse direction, either from carbon dioxide (CO_2_) or carbon monoxide (CO) to acetyl-CoA and further compounds or from acetyl-CoA and its precursors, such as acetate, towards CO_2_. Acetoclastic methanogens utilize the pathway in reverse direction generating energy by converting acetate to methane (CH_4_) and CO_2_ [39, 40]. Hydrogenotrophic methanogens use the Eastern/Methyl branch for methane formation as well as the forward direction of the Western/Carbonyl branch for cell carbon assimilation or acetate generation.

The Western/Carbonyl and the Eastern/Methyl branches of the WL pathway are nearly completely encoded in the *Bathyarchaeota* MAGs analyzed, with the exception of the genes encoding methylenetetrahydromethanopterin dehydrogenase (Mtd) and 5,10-methylenetetrahydromethanopterin reductase (Mer), which were probably missed by the binning approach. Acetyl-CoA, produced by enzymatic reactions of the WL pathway, plays an important role in the cell carbon cycle and also feeds into the TCA cycle, the genes of which are encoded in the *Bathyarchaeota* MAGs. Genes for acetate assimilation mediated by phosphotransacetylase (*pta*) and acetate kinase (*ack*) needed for conversion of acetyl-CoA to acetylphosphate and subsequently to acetate were not identified in any of the five MAGs. This is in agreement with previous findings described for the *Bathyarchaeota* MAGs BA1 and BA2 [10], but is controversial to the findings of He et al. [17] for the MAGs B24, B26-1, and B26-2. However, the acetyl-CoA synthase gene (*acd*) involved in acetate formation from acetyl-CoA and vice versa is encoded in all *Bathyarchaeota* MAGs of this study, with acetate being proposed as possible fermentation end-product (Fig. 3, Additional file 7).

### Absence of genes for enzymes involved in methanogenesis in the compiled *Bathyarchaeota* MAGs

Since *Bathyarchaeota* MAGs were recovered from metagenome sequence datasets of biogas-producing biofilms, further genes and pathways playing a role in methane metabolism were analyzed. Neither hydrogenotrophic nor acetoclastic or methylotrophic methanogenesis pathways were completely encoded in the *Bathyarchaeota* MAGs. Furthermore, the *mcr*A gene encoding for methyl-coenzyme M reductase, the key enzyme of the methane production process, is also missing in the five MAGs analyzed, indicating for incapacity of these MAG to produce methane. Additional *mcr*A gene sequence screening in the metagenome datasets leads to the identification of two *mcr*A gene sequences, showing sequence identity of 93 and 94% with uncultured archaeal clones or *Methanoculleus marisnigri*, respectively.

However, all MAGs possess complete sets of genes encoding [NiFe] membrane-bound hydrogenase (Ech), cytoplasmic coenzyme F_420_-reducing [NiFe]-hydrogenase (Frh), and cytoplasmic [NiFe]-hydrogenase (Mvh) needed for activation of H_2_ during methanogenesis. Moreover, genes encoding heterodisulfide reductase (Hdr) and cytoplasmic [NiFe]-hydrogenase (Mvh) also were identified. Likewise, almost all genes of the V-type Na^+^/H^+^-transporting ATPase (*atp*ABCDEFHIK) were also nearly completely detected in the *Bathyarchaeota* MAGs. These findings indicate that a membrane-bound electron transport chain potentially enabling energy conservation based on a proton or sodium membrane gradient and an ATPase activity may operate.

### Capacities of compiled *Bathyarchaeota* MAGs to face unfavorable process conditions

**Fig. 5:**
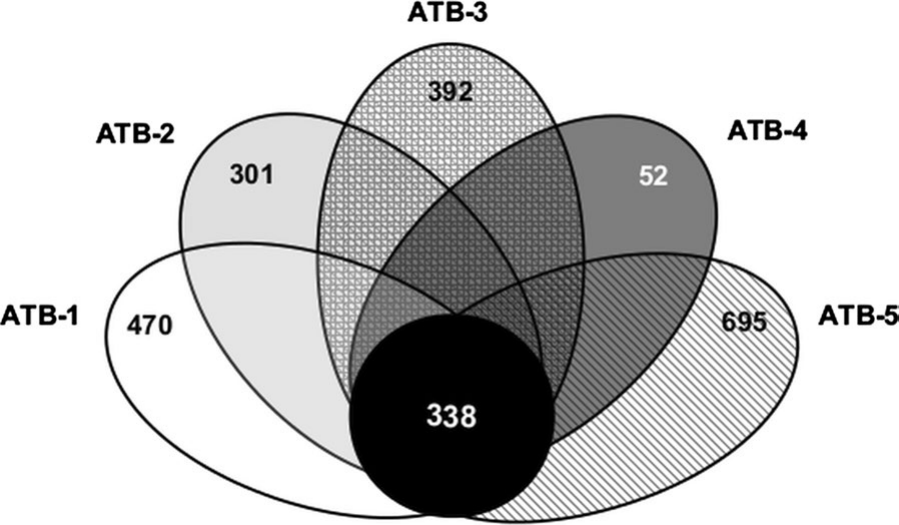
Venn diagram representing the core genome of the *Bathyarchaeota* MAGs ATB-1, -2, -3, -4, and -5. The number of orthologous coding sequences (CDSs) shared by all bins is in the center. Numbers in non-overlapping portions of each oval show the number of CDSs unique to each bin

To examine the unique metabolic potential of the five detected *Bathyarchaeota* MAGs, the MAG-specific gene sets were calculated and classified according to Cluster of Orthologous Groups of proteins (COG) categories (Additional file 8) applying the web server for metagenomic analysis WebMGA [41]. Between 52 (in case of MAG ATB-4) and 695 (in case of MAG ATB-5) singletons were found (Fig. 5). About three quarters of each MAG’s unique genes do not correlate to any gene in the COG database.

However, many COG-classified singletons represent genes for proteins participating in amino acid transport and metabolism (E), inorganic ion transport and metabolism (P), or carbohydrate transport and metabolism (G). These functional categories are of importance for AD, since they are primarily connected with plant biomass degradation.

MAG ATB-2, originating from the thermophilic AF-packing-attached methanogenic biofilm of *system 3*, possesses more classified genes than the other *Bathyarchaeota* MAGs. Among its 301 singletons are genes coding for 192 hypothetical proteins, but also for a zinc dependent phospholipase, cadmium, cobalt, and zinc antiporters, and a potassium proton pump. Hence, phospholipid degradation might play a role for the *Bathyarchaeota* taxon represented by MAG ATB-2. The presence of the potassium transporter might be involved in compensation of osmotic stress as supposed for the methanogenic archaeon *Methanoculleus bourgensis* MS2^T^ [42].

Among the other *Bathyarchaeota* MAGs, ATB-5 possesses many classified singletons (61%), representing those genetic determinants that may specify characteristic features of this MAG. These 695 MAG-specific genes encode proteins involved in transport of the amino acids leucine, isoleucine, and valine. Furthermore, genes encoding proteins for trehalose utilization as carbon or energy source and lactate synthesis mediated by lactate dehydrogenase were also identified.

Transport of ions and nutrients is of importance for microorganisms as reflected by the wide variety of encoded enzymatic pathways. Hence, the supply of anaerobic digesters converting crop material with trace elements is crucial [43]. The *Bathyarchaeota* MAGs determined in this study were screened for their coding capacity regarding transport systems for inorganic and metal ions and other compounds. Genes encoding transport systems for calcium, potassium, cadmium, magnesium, cobalt, zinc, and phosphate were identified (Additional file 7).

Furthermore, a gene encoding the archaeal-specific ammonium (NH_4_^+^) transporter (*amt*), also known from the euryarchaeon *Archaeoglobus fulgidus* [44], was identified in all MAGs except for the MAG ATB-4. NH_4_^+^ can be assimilated directly by glutamine synthetase (GS) and glutamate synthase (GOGAT) into glutamine and glutamate, respectively. The genes encoding these enzymes are present in all five analyzed *Bathyarchaeota* MAGs.

Analysis of the *Bathyarchaeota* MAGs revealed also several genes of the glyoxalase metabolism, a common pathway involved in the conversation of the toxic glycolytic byproduct methylglyoxal to d-lactate [45]. First, the glycolysis intermediate glycerone phosphate is converted to methylglyoxal by the methylglyoxal synthase (Mgs) and subsequently to the thioester S-d-lactoyltrypanothione via the enzyme glyoxalase-I (GloA). In the second step, glyoxalase-II (GloB) catalyzes hydrolysis of this thioester, releasing d-lactate. Genes encoding all three enzymes were only identified in the MAGs ATB-1, -2, -3, and -4, whereas the remaining bin ATB-5 does not encode the methylglyoxal synthase (Mgs) involved in the first reaction step of the glyoxalase metabolism.

MAG ATB-1 was the only one harboring genes of the Clustered Regularly Interspaced Short Palindromic Repeats (CRISPR) *cas* system, an adaptive microbial immune system that provides resistance against invasion of phages and mobile elements. In the MAG ATB-1, nine *cas* genes of type I-A were identified, which are located in direct vicinity to the CRISPR sequences (data not shown). The CRISPR array is composed of ten 37-bp-direct-repeats and nine spacers of 39 bp. The presence of CRISPR systems in *Bathyarchaeota* is in line with previously published findings indicating that *Archaea* may deal with foreign-DNA infections in its habitat, e.g., phages [42].

Additionally, to identify unique genes, present only in *Bathyarchaeota* members originating from biogas reactor environments, the core genome of the MAGs ATB-1 to 5 was compared with the pan genome of fourteen other *Bathyarchaeota* MAGs (for details see Fig. 2 and Additional file 2) using the program EDGAR. In total, 17 unique genes, also called singletons, were identified for the group of biogas *Bathyarchaeota* indicating that biogas biofilm *Bathyarchaeota* are not characterized by specific capabilities. The unique genes of *Bathyarchaeota* MAGs from biogas systems encode eight hypothetical proteins as well as enzymes of the amino acid synthesis metabolism.

## Conclusions

In contrast to the *Bathyarchaeota* detected in coal-bed methane wells [10], the *Bathyarchaeota* in the analyzed biogas reactor biofilms are not able to produce methane via the hitherto known methanogenesis pathway. However, the reconstruction of the metabolic pathways suggests that the analyzed *Bathyarchaeota* may base their metabolisms on carbohydrates and amino acids utilization as well as on CO_2_ fixation. Genes for extracellular hydrolysis of cellulose but also extracellular peptidases with corresponding transporter systems were found. Acetate and lactate were predicted as possible end-products of the fermentation process. Based on these findings, the analyzed MAGs were predicted to represent hydrolytic and eventually also cellulolytic and proteolytic *Archaea* involved in hydrogenesis and acidogenesis within the AD and biomethanation process. Due to their presence in biofilms, also a syntrophic co-operation with methanogenic *Euryarchaeota* could be possible. This is an outstanding finding for members of the domain *Archaea*, since only bacterial microorganisms were previously thought to be involved in the anaerobic biomass degradation in biogas reactor systems.

This study initiates rethinking of the task sharing between *Bacteria* and *Archaea* regarding successive decomposition of macromolecular compounds. Future work has to show whether findings obtained for laboratory-scale biogas reactors can be biotechnologically exploited by applying *Bathyarchaeota* species in industrial-scale biomass conversion processes. Accordingly, it is important to determine the occurrence of *Bathyarchaeota* members in industrial, i.e., production-scale biogas plants. In particular, correlations of their abundances with the utilization of specific substrates or particular reactor characteristics and conditions should be uncovered. Continuative studies will certainly benefit from the comprehensive genomic information on *Bathyarchaeota* members from biogas reactor systems by integrating this knowledge into models describing interactions within complex AD communities.

## Additional files

**Additional file 1.** Supporting information on materials and methods.

**Additional file 2.** Previously published MAGs for the phylum *Bathyarchaeota*. The phylogenetic affiliation is shown in Fig. 2. *, completeness and contamination were estimated by CheckM [25].

**Additional file 3.** Sequencing and assembly statistics of the metagenome data for the microbial communities of the thermophilic and mesophilic biogas reactor systems. UASS, upflow anaerobic solid-state reactor; AF, anaerobic filter; n.d., not determined.

**Additional file 4.** Compiled metagenome-assembled genomes that met the criteria of a minimum of 50% genome completeness and contamination rates of 10%.

**Additional file 5.** The average nucleotide identity (ANI) analysis of the analyzed *Bathyarchaeota* MAGs.

**Additional file 6.** Phylogenetic position of the *Bathyarchaeota* MAG ATB-1 within the selected archaeal representatives available in the SILVA database. The scale bar below the tree represents sequence divergence.

**Additional file 7.** Genomic loci of the analyzed *Bathyarchaeota* MAGs encoding those enzymatic functions, which are mentioned in the metabolic scheme (Fig. 3) as well as in the main text. n.d., not determined.

**Additional file 8.** Categorization of analyzed *Bathyarchaeota* MAGs’ unique genes according to Clusters of Orthologous Groups of proteins (COGs).

## Abbreviations

AD: anaerobic digestion
AF: anaerobic filter
BGP: biogas plant
MAG: metagenome-assembled genomes
CAZymes: carbohydrate-active enzymes
GH: glycosyl hydrolase
LCA: lowest common ancestor
OLR: organic loading rate
UASS: upflow anaerobic solid-state reactor
VS: volatile substances
