# Supplementary material for: Characterization of Bathyarchaeota genomes assembled from metagenomes of biofilms residing in mesophilic and thermophilic biogas reactors
Source: Biotechnol Biofuels. 2018 Jun 19;11:167. doi: 10.1186/s13068-018-1162-4 (PMC6010159; doi:10.1186/s13068-018-1162-4)
Supplement: Supplementary file 1 — Additional file 1. Supporting information on materials and methods. [file 13068_2018_1162_MOESM1_ESM.docx]

**Characterization of *Bathyarchaeota* genomes assembled from metagenomes of biofilms residing in mesophilic and thermophilic biogas reactors**

**Additional file**

Irena Maus^1,2^† (irena.maus@cebitec.uni-bielefeld.de), Madis Rumming^2,3^† (mrumming@uni-bielefeld.de), Ingo Bergmann^1^ (bergmann.i@web.de), Kathrin Heeg^1^ (kheeg@atb-potsdam.de), Marcel Pohl^4^ (marcel.pohl@dbfz.de), Edith Nettmann^5^ (edith.nettmann@rub.de), Sebastian Jaenicke^6^ (sebastian.jaenicke@computational.bio.uni-giessen.de), Jochen Blom^6^ (jochen.blom@computational.bio.uni-giessen.de), Alfred Pühler^2^ (puehler@cebitec.uni-bielefeld.de), Andreas Schlüter^2‡^ (aschluet@cebitec.uni-bielefeld.de), Alexander Sczyrba^2,3‡^ (asczyrba@techfak.uni-bielefeld.de), Michael Klocke^1‡*^ (mklocke@atb-potsdam.de)

†, ‡ Contributed equally

* Corresponding author

^1^ Dept. Bioengineering, Leibniz Institute for Agricultural Engineering and Bioeconomy, Max-Eyth-Allee 100, 14469 Potsdam, Germany

^2^ Center for Biotechnology (CeBiTec), Bielefeld University, Universitätsstrasse 27, 33615 Bielefeld, Germany

^3^ Computational Metagenomics, Faculty of Technology, Bielefeld University, Universitätsstrasse 25, 33615 Bielefeld, Germany

^4^ Biochemical Conversion Department, Deutsches Biomasseforschungszentrum gemeinnützige GmbH, Torgauer Straße 116, 04347 Leipzig, Germany

^5^ Urban Water Management and Environmental Engineering, Faculty of Civil and Environmental Engineering, Ruhr University Bochum, Universitätsstraße 150, 44780 Bochum, Germany

^6^ Dept. Bioinformatics and Systems Biology, Justus-Liebig University Gießen, Heinrich-Buff-Ring 58, 35392 Giessen, Germany

**^*^ Corresponding author**

Dr. Michael Klocke

Leibniz Institute for Agricultural Engineering and Bioeconomy (ATB), Dept. Bioengineering, Max-Eyth-Allee 100, 14469 Potsdam, Germany

Phone: +49 (0)331 5699 113

E-Mail: mklocke@atb-potsdam.de

**Reactor set-up, operation and sampling of methanogenic biofilms**

Three laboratory-scaled experimental biogas fermenter systems were sampled.

***System 1*** was a thermophilic (55 °C) two-phase, two-stage reactor system consisting of an upflow anaerobic solid-state (UASS) reactor and a downstream packed bed anaerobic filter (AF) with working volumes of 39 L and 30 L, respectively. As sole fermentation substrate, wheat straw was fed to the UASS. The straw was milled to an average cutting length of 35 mm and dedusted thereafter. On the day of sampling (day 160 of reactor operation, 2011-07-12), the organic loading rate (OLR) was 8 g volatile substances (VS) L^-1^ d^-1^, *i.e.*, 9.9 g chemical oxygen demand (COD) L^-1^ d^-1^ at a solids retention time of 3 to 7 days. The inaccuracy in retention times is caused by the batch-wise digestate removal. In the course of the experiment, an OLR of 8 g_VS_ L^-1^ d^-1^ was the lowest for the AF to outperform the UASS in terms of methane production. On the sampling day, the methane yield of the UASS-AF system was at 0.167 L g^-1^ organic dry mass (ODM), compared to an average of
0.144 L g_ODM_^-1^ over the preceding 20 days. Additionally, at this stage of the experiment, the UASS was considered as a hydrolysis stage as it only delivered 39% of the system’s methane production, as compared to 74% at OLR 6 g_VS_ L^-1^ d^-1^ or even 89% at the OLR of 2.5 g_VS_ L^-1^ d^-1^. The pH for the UASS and AF at that time was about neutral at 7.09 and 7.45, respectively. Further details on the construction, operation, reactor performance, and physico-chemical analyses were previously published [18]. Samples for microbial DNA extraction and subsequent metagenome sequencing were taken from the wheat straw digestate (cellulolytisch/hydrolytisch, phase one) and attached microbial biofilm in the UASS and were stored at -20 °C until further processing.

***System 2*** was constructed similar to *system 1* but with a working volume of 27 L for the UASS and 22 L for the AF. UASS and AF were operated at 37 °C. As fermentation substrates, maize silage was co-digested with straw in a mixture of 95 : 5 ODM at an OLR of 3.0 g_VS_ L^-1^ d^-1^. Overall biogas and methane yields were 0.61 and 0.33 L g^-1^ organic substances (OS), respectively. In the AF, the average biogas production was 9.38 L d^-1^ with an average methane content of 63.2% (v/v). Samples were taken from the biofilms on the surfaces of randomly selected polyethylene packings of the AF at day 72 of operation (2011-06-29).

***System 3*** was constructed, operated and sampled similar to *system 2* but in this case, the system was operated at 55 °C. At the day of sampling, the total VFA concentration was 0.18 g L^-1^ and 0.06 g L^-1^ for the UASS and the AF, respectively. Overall biogas and methane yields were 0.58 and 0.31 L g_OS_^-1^. In the AF, the average biogas production was 7.02 L d^-1^ with an average methane content of 61.8% (v/v). Further details on construction, operation and physico-chemical analysis of reactor *systems 2* and *3* were previously published by [19].

**High-throughput metagenome sequencing, metagenome assembly, binning and functional analyses of *Bathyarchaeota* metagenome-assembled genomes (MAGs)**

Total microbial community DNA was extracted using the FastDNA^TM^ Spin Kit for Soil (MP Biomedicals, USA) according to the manufacturer’s instructions. For sequencing purposes, 1 µg of each DNA sample was used to construct metagenome shotgun libraries applying the TruSeq DNA PCR-Free Library Preparation Kit (Illumina). Sequencing was done on the Illumina MiSeq system utilizing the V2 kit chemistry as described by the manufacturer to generate 2 × 250 bp paired-end reads.

The metagenome sequence data of the samples obtained from the bioreactor systems were trimmed and quality controlled using Trimmomatic [51]. Subsequently, pooled metagenome data (three replicates of *system 1*) have been processed for a combined assembly with megahit [20] setting the ‘meta-sensitive’ option and a minimal contig size of 1,000 bp. Likewise, metagenome data of *system 2* and *system 3* were individually processed. Mappings of the metagenome data sets onto the assemblies were performed applying bbmap from the BBTools package [21] and were further processed with samtools [22]. Genes on contigs were predicted with prodigal [52] and further aligned with diamond [53] against the NCBI nr database [54] (accessed at 2016-09-15) in more-sensitive mode. LCAs (lowest common ancestor) of the contigs were computed with MEGAN6 [23] with the minimal coverage setting and were used as taxonomic assignments. For abundance determination of the taxonomically assigned contigs, the reads per million was computed based on the mapped sequencing reads per reactor system individually.

Binning of the assemblies was performed with metabat [24] on contigs with a minimal coverage of 2-fold resulting in metagenomically assembled genomes (MAGs). From the filtered contigs taxonomically assigned as *Bathyarchaeota*, the mapped sequence reads were extracted and assembled with SPAdes [55] in careful mode. Statistics on the metabat and refined SPAdes MAGs were computed with QUAST [56]. Contamination and completeness level of the identified *Bathyarchaeota* MAGs were assessed with CheckM [25]. ACDC [26] was performed additionally to CheckM as a second contamination check. The SPAdes assembled *Bathyarchaeota* MAGs were searched with RNAmmer [57] for 16S rRNA genes.

Obtained *Bathyarchaeota* MAGs were annotated applying the program Prokka [27] and uploaded into the software platform GenDB [28] for functional analysis. Manual metabolic pathway reconstruction was carried out by means of the KEGG pathway mapping option implemented in GenDB that compares encoded gene products with corresponding protein sequences of the NCBI database (https://www.ncbi.nlm.nih.gov/), with pairwise protein sequence identities being at least 30%. To predict genes encoding carbohydrate-active enzymes, the carbohydrate-active enzyme database (CAZy) annotation web-server dbCAN [37] was used. The program EDGAR 2.0 [36], a software tool for the comparative analysis of prokaryotic genomes, was applied in order to analyze the *Bathyarchaeota* MAGs in a comparative mode. To measure the genetic and evolutionary relatedness among the MAGs, the average nucleotide identity (ANI) [33] implemented in EDGAR was used. To characterize the MAG specific genes in more detail, the corresponding amino acid sequences were functionally annotated using the RPSBLAST program on the COG database [58-59] implemented in the WebMGA web server [41] applying standard settings. The CRISPR arrays (including repeat and spacer sequences) were identified in the *Bathyarchaeota* ATB-1 by CRISPRfinder [60] as described previously [61].

**Phylogenetic classification of the analyzed *Bathyarchaeota* MAGs in relation to members of the domain *Archaea***

To phylogenetically classify the *Bathyarchaeota* MAGs analyzed in relation to members of the domain *Archaea,* the phylogenetic trees based on concatenated Single-Copy-Genes (SCG) and, in addition, on 16S rRNA genes were constructed.

The SCG phylogenetic tree was built with 14 MAGs assigned previously to the phylum *Bathyarchaeota* or to MCG (Supplementary Table S1), respectively, 128 archaeal genomes selected from IMG/M [29] as those sequenced and analyzed at the DOE Joint Genome Institute, and *Ruminiclostridium thermocellum* ATCC 27405 as outgroup. Amino acid sequences from 37 encoded SCG marker motifs from phylosift [46] were called on the whole dataset, and for each SCG motif, a multiple sequence alignment (MSA) was computed with MUSCLE [47]. The final SCG-based phylogenetic tree was computed using RAxML Version: 8.1.16 [30] with concatenated MSAs as input and visualized with Phyl.io [31].

The 16S rRNA gene based tree was generated using 16S rRNA gene sequences derived from selected archaeal representatives publically available in the SILVA database. Calculation of this phylogenetic tree was also accomplished applying RAxML and visualized with Phyl.io.
