# Supplementary figures and images for: Characterization of Bathyarchaeota genomes assembled from metagenomes of biofilms residing in mesophilic and thermophilic biogas reactors
Source: Biotechnol Biofuels. 2018 Jun 19;11:167. doi: 10.1186/s13068-018-1162-4 (PMC6010159; doi:10.1186/s13068-018-1162-4)

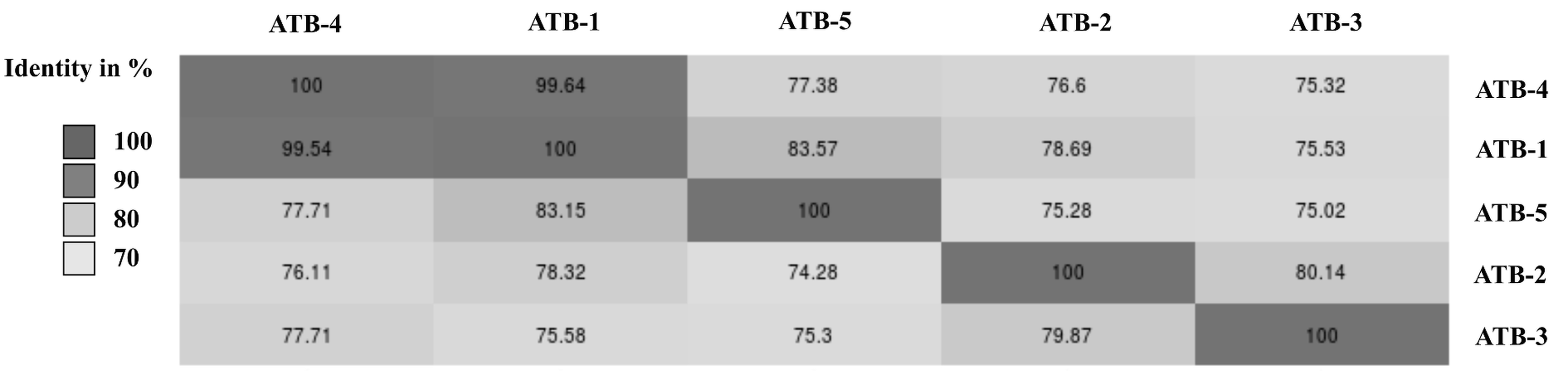

Supplement: Supplementary file 5 — Additional file 5. The average nucleotide identity (ANI) analysis of the analyzed Bathyarchaeota MAGs. [file 13068_2018_1162_MOESM5_ESM.tiff]

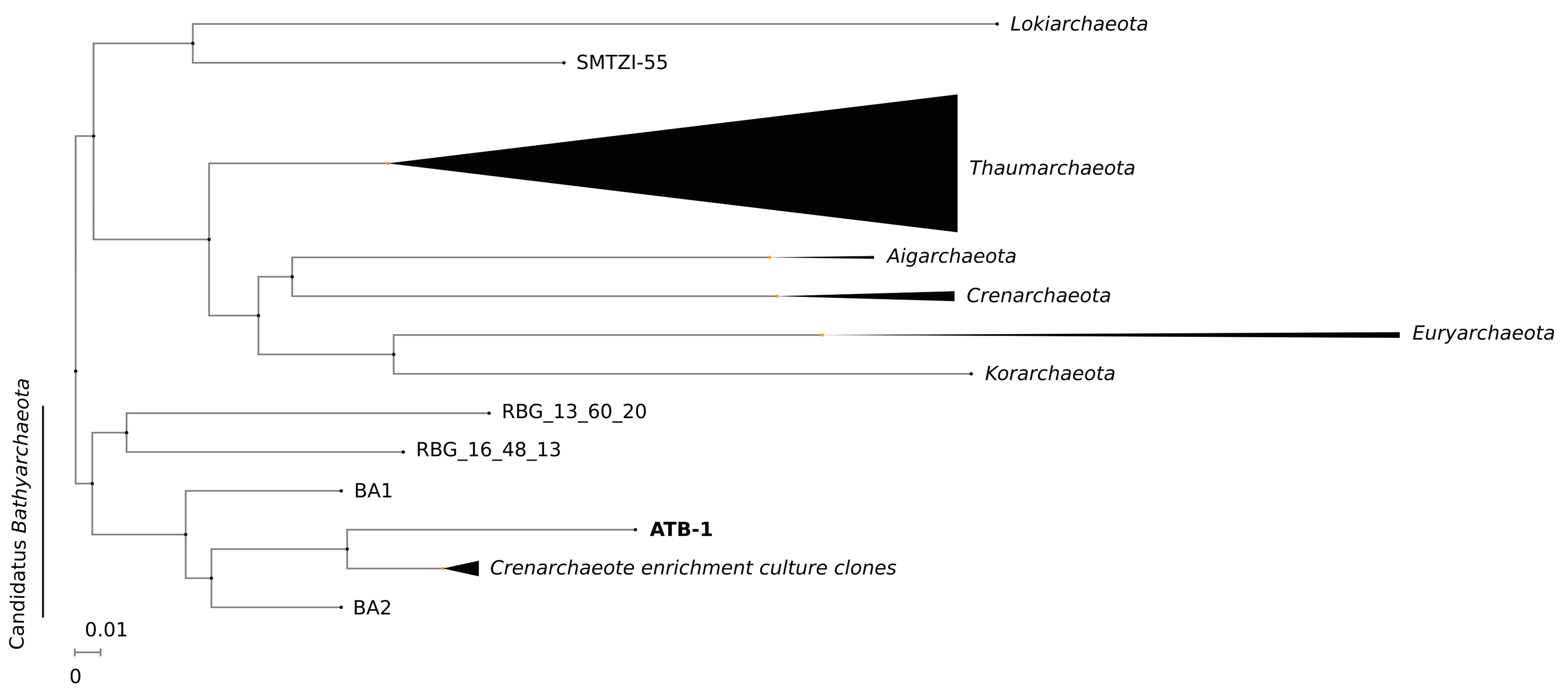

Supplement: Supplementary file 6 — Additional file 6. Phylogenetic position of the Bathyarchaeota MAG ATB-1 within the selected archaeal representatives available in the SILVA database. The scale bar below the tree represents sequence divergence. [file 13068_2018_1162_MOESM6_ESM.tiff]

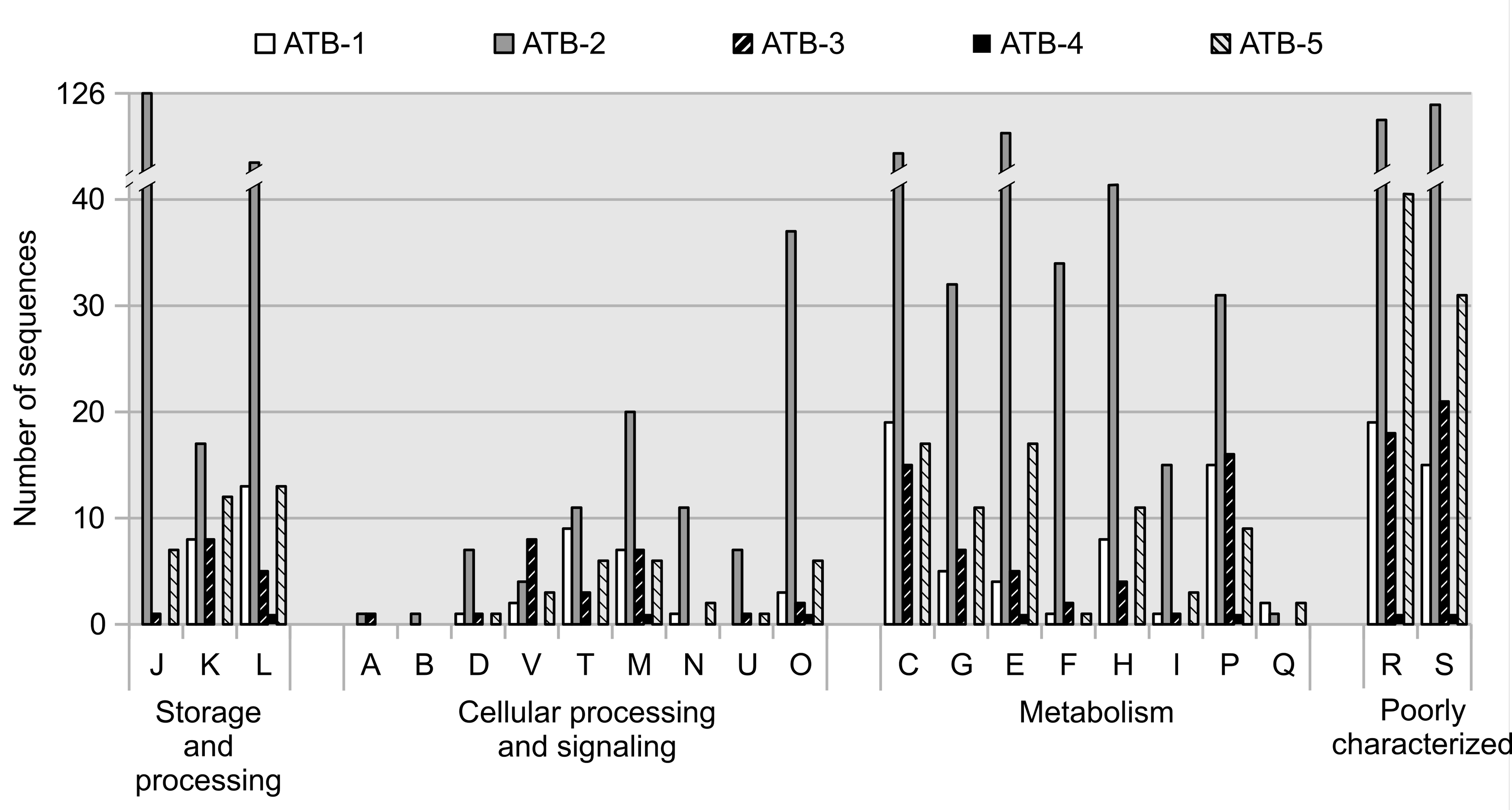

Supplement: Supplementary file 8 — Additional file 8. Categorization of analyzed Bathyarchaeota MAGs’ unique genes according to Clusters of Orthologous Groups of proteins (COGs). [file 13068_2018_1162_MOESM8_ESM.tiff]
